# Missense mutation of *NRAS* is associated with malignant progression in neurocutaneous melanosis

**DOI:** 10.1186/s40478-024-01723-0

**Published:** 2024-01-22

**Authors:** Haruhiko Takahashi, Manabu Natsumeda, Norikazu Hara, Akihide Koyama, Hiroshi Shimizu, Akinori Miyashita, Daiken Satake, Yoshihiro Mouri, Jun Tsukano, Keita Kawabe, Yoshihiro Tsukamoto, Masayasu Okada, Ryosuke Ogura, Akihiko Yuki, Hajime Umezu, Akiyoshi Kakita, Takeshi Ikeuchi, Makoto Oishi

**Affiliations:** 1 Department of Neurosurgery, Brain Research Institute, Niigata University, 1-757 Asahimachi, Chuo-ku, 951-8585 Niigata, Japan; 2 Advanced Treatment of Neurological Diseases Branch, Brain Research Institute, Niigata University, 1- 757 Asahimachi, Chuo-ku, 951-8585 Niigata, Japan; 3 Department of Molecular Genetics, Brain Research Institute, Niigata University, 1-757 Asahimachi, Chuo-ku, 951-8585 Niigata, Japan; 4 Department of Legal Medicine, Graduate School of Medical and Dental Science, Niigata University, Niigata University, 1-757 Asahimachi, Chuo-ku, 951-8510 Niigata, Japan; 5 Department of Pathology, Brain Research Institute, Niigata University, 1-757 Asahimachi, Chuo-ku, 951-8585 Niigata, Japan; 6 Division of Dermatology, Graduate School of Medical and Dental Sciences, Niigata University, Niigata University, 1-757 Asahimachi, Chuo-ku, 951-8510 Niigata, Japan; 7 Division of Pathology, Niigata University Medical and Dental Hospital, Niigata University, 1-754 Asahimachi, Chuo-ku, 951-8510 Niigata, Niigata, Japan

**Keywords:** Droplet digital polymerase chain reaction, Multiregional genomic analysis, Neurocutaneous melanosis, Whole exome sequencing

## Abstract

Neurocutaneous melanosis (NCM) is a rare congenital neurocutaneous syndrome characterized by congenital melanocytic nevus of skin and abnormal proliferation of leptomeningeal melanocytes. Early acquisition of post-zygotic somatic mutations has been postulated to underlie the pathogenesis of NCM. The pathogenesis of NCM remains to be fully elucidated, and treatment options have not been established. Here, we report for the first time, multiregional genomic analyses in a 3-year-old autopsied girl with leptomeningeal melanomatosis associated with NCM, in which a ventriculo-peritoneal (VP) shunt was inserted for the treatment of hydrocephalus. The patient expired six months after the onset due to respiratory failure caused by abdominal dissemination via VP shunt. We performed multiregional exome sequencing to identify genomic differences among brain and abdominal tumors, nevus, and normal tissues. A total of 87 somatic mutations were found in 71 genes, with a significantly large number of gene mutations found in the tumor site. The genetic alterations detected in the nevus were only few and not shared with other sites. Three mutations, namely *GNAQ* R183Q, *S1PR3* G89S and *NRAS* G12V, considered pathogenic, were found, although *S1PR3* mutations have not been previously reported in melanocytic tumors. *GNAQ* and *S1PR3* mutations were shared in both tumor and normal sites. Moreover, the mutant allele frequencies of the two mutations were markedly higher in tumor sites than in normal sites, with copy-neutral loss-of-heterozygosity (CN-LOH) occurring in tumor. *NRAS* mutation was found only in the abdominal tumor and was thought to be responsible for malignant progression in the present case. Multiregional comprehensive genetic analysis may lead to discovering novel driver mutations associated with tumorigenesis and targeted therapy.

**Supplementary Information:**

The online version contains supplementary material available at 10.1186/s40478-024-01723-0.

## Introduction

Neurocutaneous melanosis (NCM) is a rare congenital neurocutaneous syndrome characterized by large and/or multiple congenital melanocytic nevi (CMN) of skin and abnormal proliferation of leptomeningeal melanocytes [3]. Early acquisition of post-zygotic somatic mutations has been postulated to underlie the pathogenesis of NCM; common genetic alterations have been described in CMN and central nervous system (CNS) lesions [6, 9, 14]. Although dormant cells of CMN are histologically benign, CNS lesions can become malignant in childhood or the second or third decades, and the prognosis of NCM patients is dismal. Despite recent advances in molecular genetics, the pathogenesis of NCM remains to be fully elucidated, and treatment options have not been established. To detect true driver mutations associated with tumorigenesis and progression, we sought to elucidate genetic differences between CMN and tumors in a single case. Comprehensive genetic analysis of CMN and CNS lesions may lead to discovering novel driver mutations associated with tumorigenesis and progression, revealing new targeted strategies.

In the current study, we report for the first time, multiregional genomic analyses in a patient with leptomeningeal melanomatosis associated with NCM, in which a ventriculo-peritoneal (VP) shunt was inserted for the treatment of hydrocephalus. We found an additional pathogenic *NRAS* mutation in the disseminating intraperitoneal tumor obtained at autopsy.

## Case presentation

**Fig. 1:**
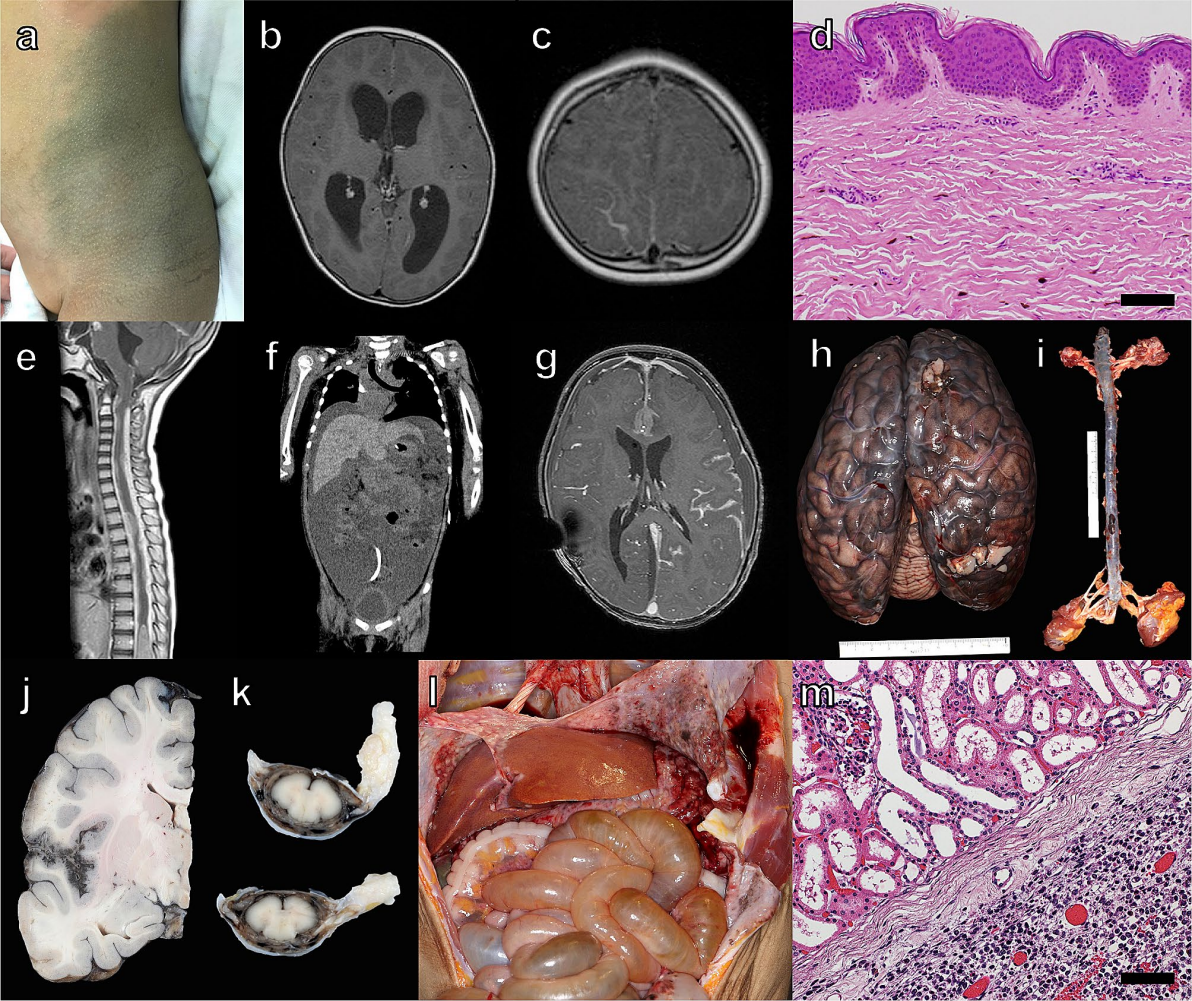
Clinicopathologic features of the patient. Photograph of the congenital cutaneous nevus **(a)**. Preoperative brain MR images **(b, c)**. Histology of the nevus **(d)**. Spinal cord MR image obtained at emergent admission for quadriplegia **(e)**. Body CT image at respiratory failure **(f)**. Brain MR image 10 days before death **(g)**. Photographs of the brain **(h)**, spinal cord **(i)**, and abdomen **(l)** at autopsy. Photographs of cut sections of the brain **(j)** and spinal cord **(k)** after formalin fixation. Histology of the kidney **(m)** Photograph of a large melanocytic nevus on the patient’s left flank to back **(a)**. Post-contrast MR images showed hydrocephalus **(b)** and linear contrast-enhanced lesion along the sulcus at the right parietal lobe **(c)**. A small number of melanocytes were scattered in the dermal layer **(d)**. Post-contrast MR image revealed extensive spinal dissemination **(e)**. Body CT showed massive ascites and compression of the lungs due to elevation of the diaphragm **(f)**. Post-contrast MR image showed an enlarged linear contrast-enhanced lesion along the brain surface and an intraparenchymal mass lesion on the corpus callosum, but no signs of cerebral herniation **(g)**. At autopsy, macroscopic examination of the whole brain and spinal cord revealed a thickened and black leptomeninges **(h, i)** and multiple masses of tumor cells on the surface of abdominal organs **(l)**. Tumor cells invaded the brain parenchyma adjacent to the Sylvian fissure **(j)** and covered the whole circumference of the spinal cord **(k)**. The tumor cells were observed on the surface of the kidney but did not infiltrate the renal medulla due to Gerota’s fascia **(m)**. Scale bars: 100 μm

A previously healthy 2-year-old girl presented with severe headaches and nausea due to acute hydrocephalus and was admitted to an affiliate hospital. She had no family history of note. Examination of the body revealed a large congenital cutaneous nevus of her left flank to back (Fig. 1a). Post-contrast MR images showed a linear contrast-enhanced lesion along the sulcus at the right parietal lobe (Fig. 1b, c). The patient was transferred to the Department of Neurosurgery, Niigata University Medical and Dental Hospital because of progressive deterioration of consciousness over a couple of days and immediately underwent cerebral spinal fluid (CSF) reservoir implantation. The brain surface observed through the burr-hole was obviously black-colored. These findings suggested her clinical diagnosis to be NCM. Intermittent drainage from the CSF reservoir failed to control the increased intracranial pressure symptoms, and a VP shunt was performed the next day. Although tissue biopsies of black brain tissue harvested from burr hole and nevus (Fig. 1d, Additional file 1: Fig. S1a) were both benign, follow-up MR images taken three weeks later showed enlargement of leptomeningeal contrast-enhancing lesions. An open biopsy was performed for targeting contrast-enhanced lesions at the right parietal lobe. Histopathological diagnosis was meningeal melanomatosis with round to spindle-shaped tumor cells with nuclear atypia invading the subarachnoid space on the brain surface; MIB-1 labeling index was 10.3% (Additional file 1: Fig. S1b-f). Her neurologic symptoms did not worsen, but follow-up MR images showed that the contrast-enhancing lesions extended to the leptomeninges on the surface of the brainstem and cranial nerve. Three weeks after surgery, whole brain irradiation (36 Gy/20 fractions) was performed, followed by nivolumab administration. A month and a half after radiotherapy, she was admitted emergently with quadriplegia. MR images showed that the intracranial lesions had not enlarged, but extensive spinal dissemination was found from the cervical cord to the cauda equina outside of the radiation field (Fig. 1e). Nivolumab administration was discontinued after 3 cycles and whole spinal cord irradiation (30 Gy/10 fractions) was commenced.However, respiratory failure due to phrenic nerve palsy progressed rapidly, and she was placed on a ventilator after undergoing a tracheotomy. Her abdominal distention progressively worsened, and CT showed massive ascites (Fig. 1f). Suspecting abdominal dissemination via the VP shunt tube, the intraperitoneal tube was removed from the abdominal cavity and connected to a continuous drainage system. CSF cytology examinations were performed several times, but no tumor cells were detected. Albumin and furosemide were administered for treating ascites, but the intracranial contrast-enhanced lesions enlarged on MR images (Fig. 1g), and her general condition worsened. She was responsive until the day before her death, six months after the onset, and a general autopsy was performed. Autopsy imaging showed massive ascites, pneumonia, and atelectasis but no signs of brain herniation.

At autopsy, macroscopic examination of the whole brain and spinal cord revealed thickened and black leptomeninges (Fig. 1h, i), and subarachnoid space was largely filled with tumor cells. The tumor cells infiltrated into the cerebral and spinal parenchyma via Virchow-Robin space or otherwise constituted masses in bilateral frontal lobes and left temporal lobe (Fig. 1j, k), Additional file 1: Fig. S1g-i). Multiple masses of tumor cells on the surface of abdominal organs, resulting from dissemination via VP shunt tube, were observed (Fig. 1l). Numerous tumor cells infiltrated most of the abdominal organs, including the liver, stomach, omentum, diaphragm, and ovaries (Additional file 1: Fig. S2), but notably, no invasion of the renal parenchyma was observed owing to the barrier of the Gerota’s fascia (Fig. 1m).

## Materials and methods

### Pathological analysis

The present study was conducted after approval from the Institutional Review Board of Niigata University (approval #G2023-0003). Informed consent for collecting samples during surgery and autopsy and their subsequent use for genetic analysis were obtained from the patient’s family.

The surgical and autopsy specimens were fixed with 10% buffered formalin and embedded in paraffin. Histopathological diagnosis was performed on 4-µm thick sections stained with hematoxylin (HE). The pathological diagnosis was made based on the 2021 WHO classification of tumors of the CNS by experienced neuropathologists (AK and HS). Immunohistochemistry was performed as described previously using primary antibodies against Ki-67 (1:100, monoclonal, clone MIB-1, DAKO, Glostrup, Denmark), HMB-45 (1:100, DAKO, Glostrup, Denmark), vimentin (1:400, DAKO, Glostrup, Denmark), S100 (1:3200, DAKO, Glostrup, Denmark) [11, 13].

### Multiregional genomic analysis

During autopsy, we cryopreserved various tissues, including tumors, nevus, and normal tissue. The nevus was preserved during the initial VP shunt surgery. Brain and abdominal tumor, cerebral cortex and white matter, normal skin, nevus, and kidney (renal medulla) were subjected to multisampling. Genomic DNA was extracted from fresh frozen tissue using the QuickGene-Auto240L (Kurabo Industries, Osaka, Japan) according to the manufacturer’s instructions.

### Whole exome sequencing

The extracted genomic DNA was then used to prepare exome libraries using the SureSelect Human All Exon V6 kits (Agilent Technologies, Santa Clara, CA, USA). Sequencing was performed on Illumina’s NovaSeq 6000 sequencer in 151-cycle paired-end mode. Sequenced reads were processed using *fastp* version 0.19.5 with default settings for quality control and adapter trimming. The cleaned reads were then mapped to the human reference genome hg38 using *BWA-MEM* 0.7.15-r1140 with default settings. Subsequent read-processing steps followed the GATK4 Best Practice recommendations [22]. Somatic short variants, including single nucleotide variants (SNV) and indels, were called using *Strelka2* v2.9.10, along with the *Manta* v1.6.0 structural variant and indel caller, according to *Strelka*’s user guide [5]. The kidney sample was used as a matched normal sample when calling somatic variants. Variants were annotated using Ensembl *VEP* release 102 and converted to a maf format through *vcf2maf* v1.6.21. We prioritized protein-altering variants that passed the variant filter assigned by *Strelka2* and visualized them using *maftools* v2.6.05 [10].

The impact of somatic mutations detected in whole exome sequencing (WES) was performed and analyzed with SIFT [12] and Polyphen [1], which predict protein function brought by amino acid changes.

### Droplet digital polymerase chain reaction

Droplet digital polymerase chain reaction (ddPCR) reagents and self-designed Primer/Probe mix for *GNAQ* R183Q, *S1PR3* G89S, and *NRAS* G12V were purchased from Integrated DNA Technologies Inc (Coralville, IA, USA) (Additional file 2: Table S1). A 20 µL aliquot of PCR mix (Final conc. Primer; 900 nM, Probe; 250 nM), composed of 5 µL of ddPCR Multiplex Supermix (Cat. No. 12,005,910, Bio-Rad, Hercules, CA, USA), 1 µL of 1 ng/uL DNA was loaded into each sample of well of an 8-channel disposable droplet generator cartridge (DG8 Cartridges and Gaskets, Cat. No. 1,864,007, Bio-Rad). An additional 60 µL of droplet generation oil (Droplet Generator Oil for Probes, Cat. No. 1,863,005, Bio-Rad) was loaded into the oil well for each channel. After droplet generation, droplets were transferred to a 96-well PCR plate and subjected to thermal cycling. Thermal cycling conditions were as follows: 95˚C 10 min, 94˚C 30 Sect. 60˚C 30 s. (for 40 cycles) 98˚C 10 min. and hold 4˚C. Amplification of the 20 µL reaction mixture was carried out on a MiniAmp™ Plus thermal cycler (Applied Biosystems). After PCR, the 96-well PCR plate was transferred to a QX-200™ droplet reader (Bio-Rad), and the data were analyzed using QX Manager 1.2 standard edition software (Bio-Rad). Mutation-specific signals of *GNAQ* R183Q, *S1PR3* G89S, and *NRAS* G12V were generated in the FAM channel, whereas wildtype signals were generated in the HEX channel. Mutant alleles frequency (MAF) was calculated as follows: MAF% = (Nmt/(Nmt + Nwt)x100), where Nmt is the number of mutant droplets and Nwt is the number of wildtype droplets per reaction.

### Allele-specific copy number analysis

Allele-specific copy number analysis was conducted using *cnv_facets* v0.16.0 with default settings [17]. Consistent with the approach for short variant discovery, the kidney sample served as a matched normal control. A pair of tumor and normal exome data were analyzed to identify somatic copy number variations (CNVs) within the exonic regions.

## Results

### Whole exome sequencing

**Fig. 2:**
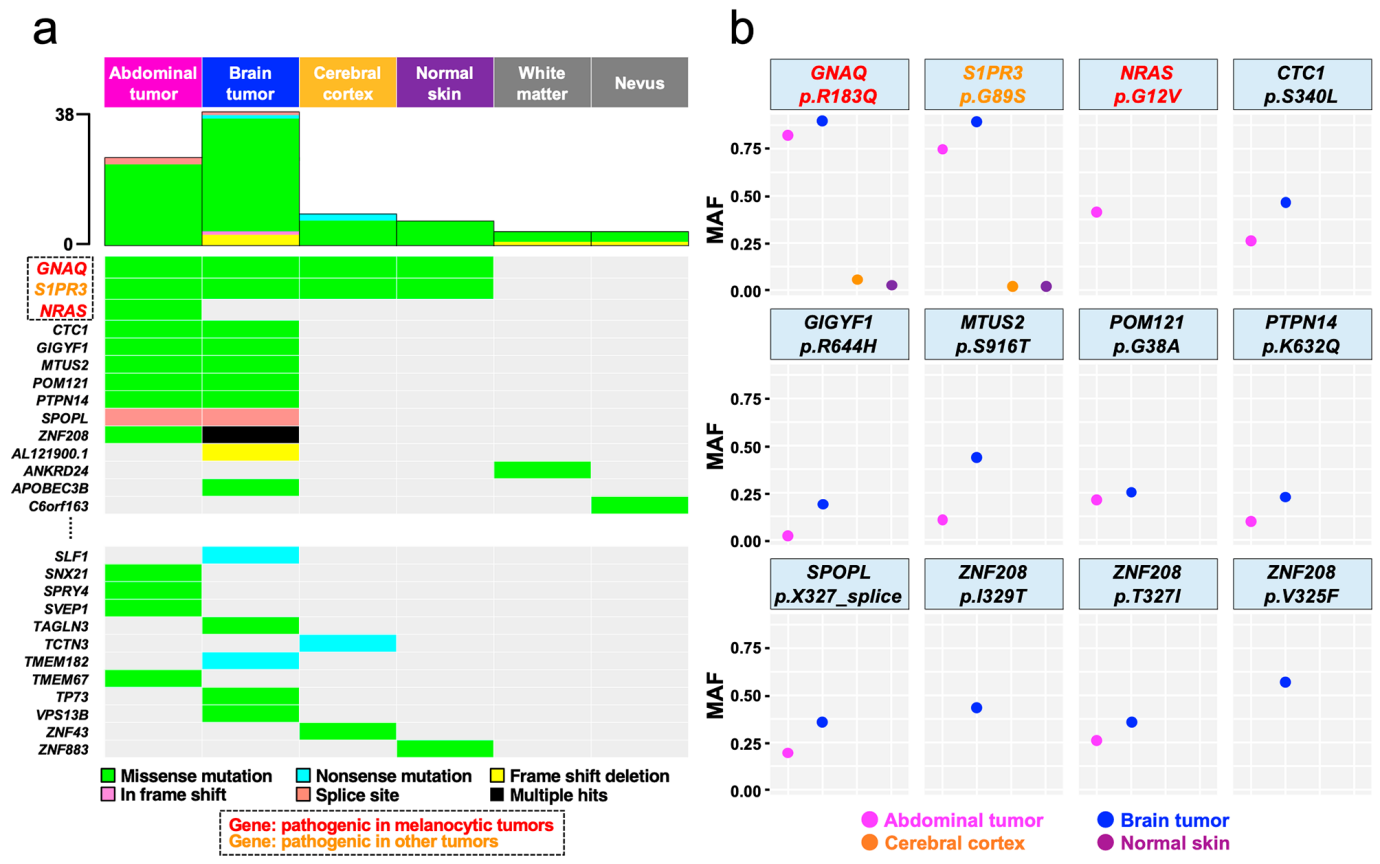
Sharing of somatic mutation profiles defined by multiregional whole exome sequencing. Sharing pattern and types of somatic mutations among six tissues **(a)**. Three somatic mutations, *GNAQ*, *S1PR3,* and *NRAS*, were determined to be pathogenic. *GNAQ* and *NRAS* (listed in red) have been reported to be associated with tumorigenesis in melanocytic tumors. On the other hand, *S1PR3* (listed in orange) has been reported to be associated with cancer in other sites but not reported in melanocytic tumors. Distribution of MAF of somatic mutations shared among brain and abdominal tumors **(b)**. *GNAQ* and *S1PR3* were shared among brain and abdominal tumors, while *NRAS* was detected only in the abdominal tumors. *Abbreviations*: MAF: mutant allele frequency

The average sequencing depth and the percentage of the exome that covered at least 20 reads were, on average, 168.7 ± 21.4 (range: 128.1–189.8) and 96.5 ± 0.61% (range: 95.1 − 97.0%), respectively, supporting confident variant analysis. A total of 87 somatic mutations in 71 genes, 81 SNV and 6 indels were detected in 6 sites (median, 8; range, 4–38), with a large number of somatic mutations found in the tumor site (Fig. 2a, Additional file 2: Table S2). On the other hand, the genetic alterations detected in the nevus were only few and not shared with other sites. The genetic alterations shared among brain and abdominal tumors are listed in Table 1. Among these, the likely pathogenic somatic mutations, *GNAQ* R183Q and *S1PR3* G89S were found in four sites: brain and abdominal tumors, cerebral cortex, and normal skin, although *SIPR3* mutations have not been previously implicated in melanocytic tumors. Moreover, the MAF of the two was apparently higher in tumor sites than in normal sites (Fig. 2b). These two genetic alterations were determined to be pathogenic according to both SIFT and Polyphen. As for others, *CTC*, *GIGYF1*, *MTUS2*, *POM121*, *PTP14*, *SPOPL*, and *ZNF 208* were detected only in tumor tissue, and none were determined to be pathogenic by both algorithms concordantly. *NRAS* G12V, a known pathogenic driver of pediatric meningeal melanomatosis associated with meningeal melanosis, was found only in the abdominal tumor and was thought to be responsible for malignant progression in the present case.

**Table 1:** Pathogenic drivers and other genetic alterations shared between brain and abdominal tumors

Gene	Chromosome	Variant Classification	HGVSc	HGVSp	Site	Number of mutant reads	Number of normal reads	MAF (%)	Significance (SIFT)	Significance (PolyPhen)	Exon (Intron)
*GNAQ*	chr9	missense mutation	c.548G > A	p.Arg183Gln	Abdominal tumor	215	47	82.1	**deleterious (0.03)**	**probably damaging (0.958)**	4/7
					Brain tumor	194	23	89.4			
					Cerebral cortex	11	207	5			
					Normal skin	7	225	3			
*S1PR3*	chr9	missense mutation	c.265G > A	p.Gly89Ser	Abdominal tumor	336	117	74.2	**deleterious (0.02)**	**possibly damaging (0.796)**	1/1
					Brain tumor	222	26	89.5			
					Cerebral cortex	13	508	2.5			
					Normal skin	10	492	2			
*NRAS*	chr1	missense mutation	c.35G > T	p.Gly12Val	Abdominal tumor	90	136	39.8	**deleterious (0)**	**possibly damaging (0.454)**	2/7
*CTC1*	chr17	missense mutation	c.1019C > T	p.Ser340Leu	Abdominal tumor	124	341	26.7	tolerated (0.68)	benign (0.158)	6/23
					Brain tumor	105	116	47.5			
*GIGYF1*	chr7	missense mutation	c.1931G > A	p.Arg644His	Abdominal tumor	12	478	2.4	**deleterious (0)**	benign (0.025)	19/27
					Brain tumor	93	383	19.5			
*MTUS2*	chr13	missense mutation	c.2747G > C	p.Ser916Thr	Abdominal tumor	31	264	10.5	tolerated (0.08)	**possibly damaging (0.621)**	6/16
					Brain tumor	53	71	42.7			
*POM121*	chr7	missense mutation	c.113G > C	p.Gly38Ala	Abdominal tumor	61	214	22.2	tolerated (0.06)	benign (0.145)	1/13
					Brain tumor	92	274	25.1			
*PTPN14*	chr1	missense mutation	c.1894A > C	p.Lys632Gln	Abdominal tumor	134	1457	8.4	tolerated (0.13)	benign (0.255)	13/19
					Brain tumor	191	636	23.1			
*SPOPL*	chr2	splice site	c.981-2A > T		Abdominal tumor	26	105	19.8			(9/10)
					Brain tumor	36	64	36			
*ZNF208*	chr19	missense mutation	c.986T > C	p.Ile329Thr	Brain tumor	11	15	42.3	tolerated (1)	benign (0.003)	4/4
*ZNF208*	chr19	missense mutation	c.980C > T	p.Thr327IIe	Abdominal tumor	16	44	26.7	tolerated (0.43)	benign (0.015)	4/4
					Brain tumor	11	21	34.4			
*ZNF208*	chr19	missense mutation	c.973G > T	p.Val325Phe	Brain tumor	21	16	56.8	tolerated (0.77)	**possibly damaging (0.632)**	4/4

Pathogenic/possibly pathogenic alterations are indicated in bold. HGVSc: Human Genome Variation Society coding DNA; HGVSp: Human Genome Variation Society protein; SIFT: Sorting Intolerant From Tolerant MAF: mutant allele frequency

### Droplet digital PCR

**Fig. 3:**
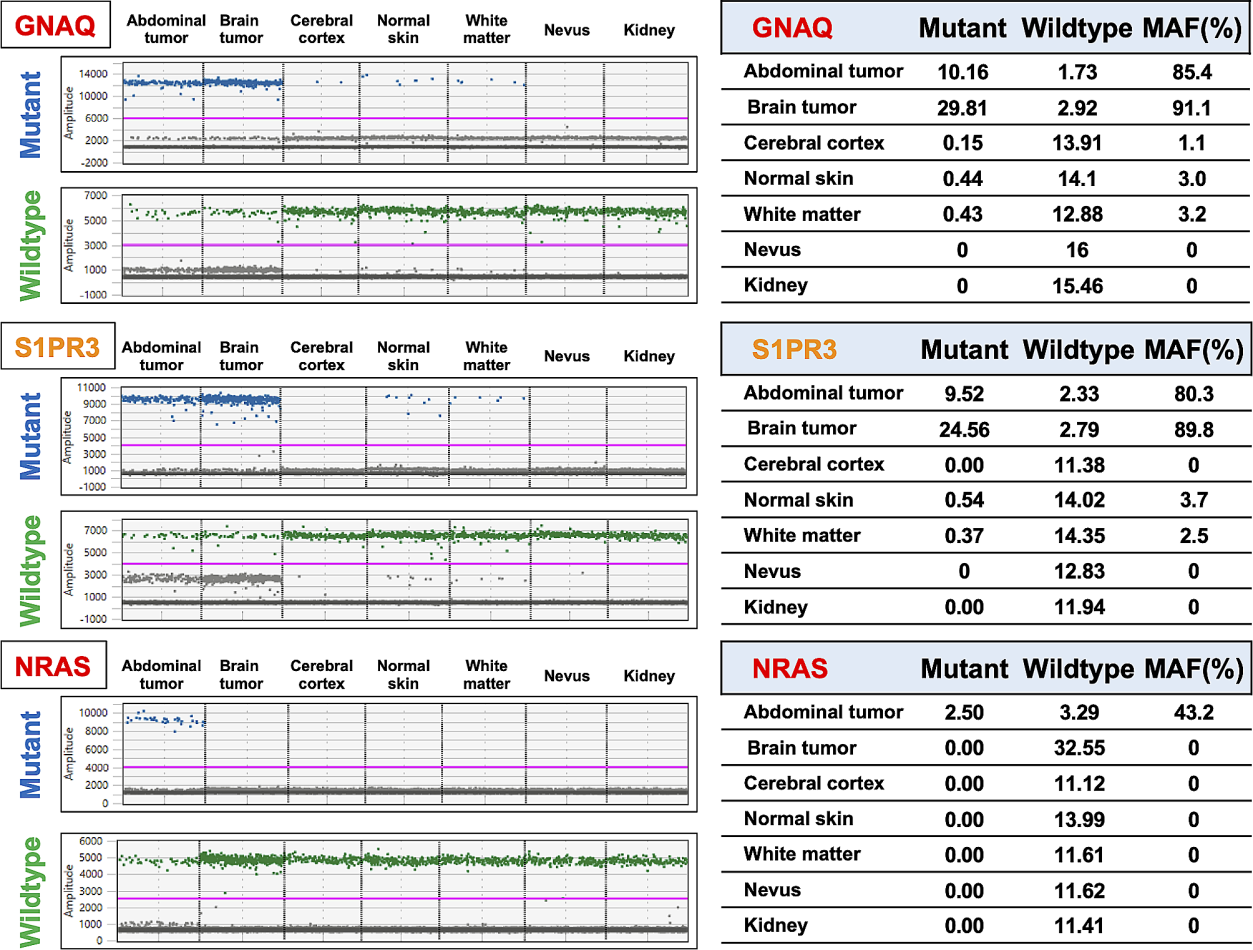
Sharing of somatic mutation profiles defined by multiregional ddPCR. *GNAQ* and *S1PR3* showed similar results, with numerous mutant droplets detected in brain and abdominal tumors. On the other hand, *NRAS* mutation was only detected in the abdominal tumor. *Abbreviations*: ddPCR: droplet digital polymerase chain reaction, MAF: mutant allele frequency

To validate the results of WES, for each site, *GNAQ* R183Q, *S1PR3* G89S, and *NRAS* G12V status were analyzed by droplet digital PCR (ddPCR) (Fig. 3). Wildtype and/or mutant droplets were detected in all samples in various frequencies. *GNAQ* and *S1PR3* showed similar results, with numerous mutant droplets detected in brain and abdominal tumors. Consistent with WES results, a small number of mutant droplets were detected in the cerebral cortex, white matter, and normal skin, while none were detected in the nevus. On the other hand, *NRAS* G12V mutation was detected only in the abdominal tumor and not from other sites.

### Allele-specific copy number analysis

**Fig. 4:**
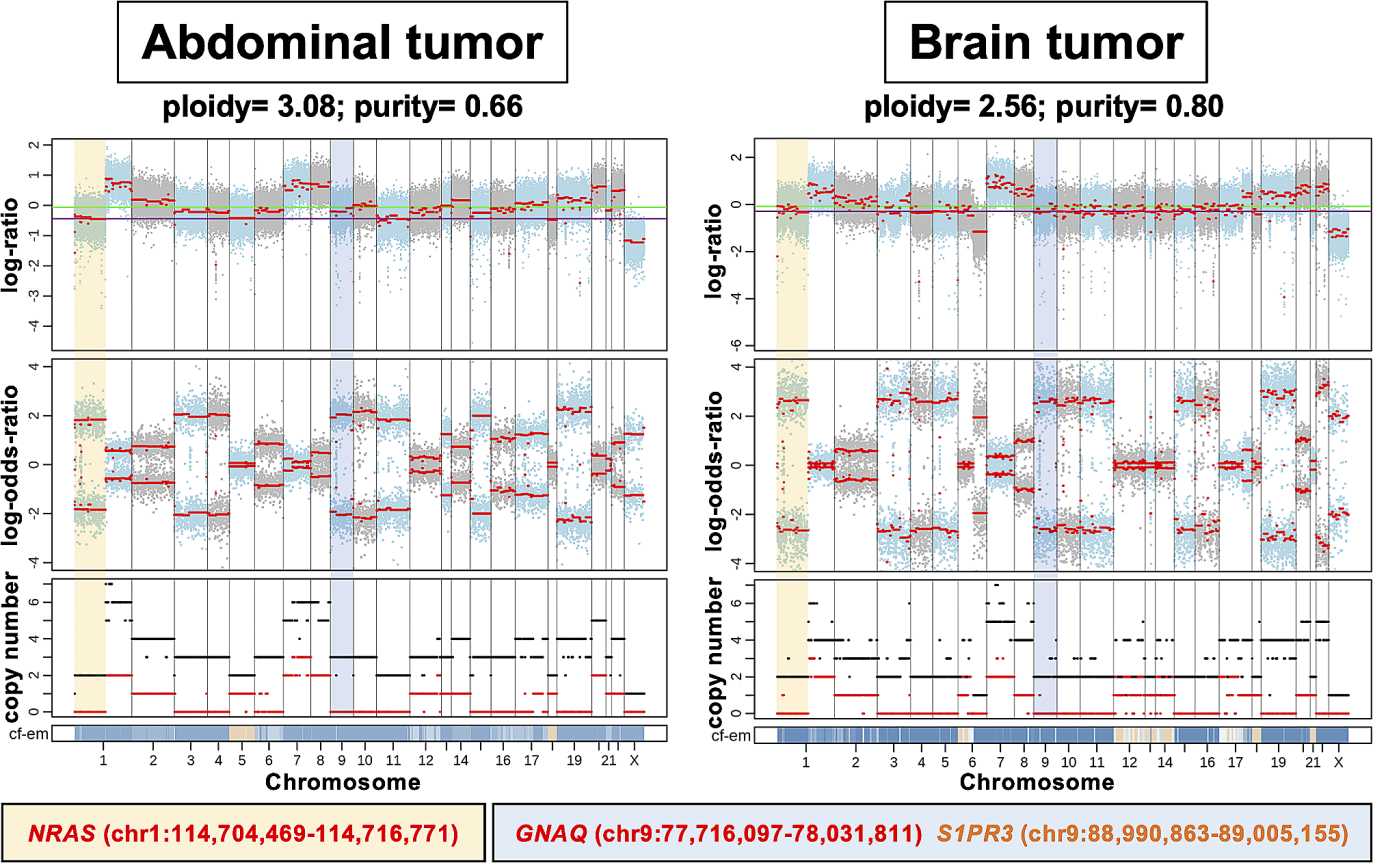
Integrated visualization of FACETS analysis of whole-exome sequencing data in abdominal and brain tumors. The top panel of the figure displays the total copy number logratio (log R). The green line indicates the median log R in the sample. The purple line indicates the log R of the diploid state. The second panel displays allele-specific log-odds-ratio (log OR). Segment means are plotted in red lines. The third panel plots the total (black) and minor (red) copy numbers for each segment. The bottom bar shows the associated cellular fraction (cf.). Dark blue indicates high cf. Light blue indicates low cf. Beige indicates a normal segment (total = 2, minor = 1)

Having determined multiregional differences in MAF of likely pathogenic mutations, we next sought to look at differences in CNVs among the different regions. The ploidy and purity of the abdominal and brain tumor were 3.08 and 0.66, 2.56 and 0.80, respectively. CNV events affecting these three likely pathogenic mutations, *GNAQ* R183Q, *S1PR3* G89S, and *NRAS* G12V, were observed exclusively in abdominal and brain tumor samples and not detected in the cerebral cortex, white matter, or skin (Fig. 4, Additional file 1: Fig. S3). We speculate that CNVs could not be detected in these samples because the proportion of tumor cells was too small. All CNV events exhibited copy-neutral loss-of-heterozygosity (CN-LOH), which is characterized by the deletion of one allele concurrent with the duplication of another homologous allele. This process results in homozygosity derived from two copies of the duplicated allele. For the abdominal tumor sample, an additional duplication of the LOH allele occurred in the chromosome 9 region, leading to a total of three copies in that specific region (Additional file 2: Table S3).

## Discussion and conclusions

We report for the first time multiregional comprehensive genomic analyses, including tumor, nevus, and normal tissue, in an autopsied NCM patient and showed *NRAS* G12V as the second driver mutation associated with malignant progression. Primary melanocytic tumors of the CNS are derived from melanocytes originating from the neural crest early during embryogenesis. Melanoblasts (precursors of melanocytes) migrate during embryonic development and travel to the skin. Most melanoblasts first reach the dermis, and smaller numbers of melanoblasts travel to mucosal surfaces (i.e., the aerodigestive and urogenital tract) and rarely to the inner ear, uvea and the leptomeninges [18]. If melanoblasts travel to the leptomeninges and melanocytes proliferate excessively, they can cause primary melanocytic tumors of the CNS. Primary melanocytic tumors of the CNS occur in adults as well as children, the latter often in the context of NCM [8].

Primary melanocytic tumors of the CNS are extremely rare and consist of benign melanocytomas and malignant melanomas. Intracranial melanomas showing diffuse invasion of the leptomeninges by malignant melanocytic cells are called primary meningeal melanomatosis. Symptomatic meningeal melanomatosis is associated with an extremely poor prognosis [3], with a reported median overall survival of only 5 months in a classic series [4]. Brain and leptomeningeal metastases of cutaneous melanomas are generally refractory to traditional chemoradiotherapy [15]. New treatments such as combination BRAF inhibitor and MEK inhibitor [16] and immune checkpoint inhibitors [19] have been advocated for progressive or metastatic cutaneous melanoma. However, recent studies have shown that primary melanocytic tumors of the CNS share common mutations with uveal melanomas, such as *GNAQ* and *GNA11* mutations, and that *BRAF* V600E mutations are seldomly found [7, 21]. Additionally, lower PD-L1 expression has been reported in metastatic uveal melanoma compared to metastatic cutaneous melanoma [2]. On the other hand, *S1PR3* has been reported to be associated with tumorigenesis in lung cancer and renal cell carcinoma [20, 23] but has not been reported in melanocytic tumors.

Allele-specific copy number analysis has major advantages over conventional total copy number analysis. It provides a much more comprehensive identification of CNV, including CN-LOH events not detectable by analyzing the total copy number alone [17]. In the present case, the MAF of *GNAQ* mutation was much higher than that of *NRAS*, probably due to CN-LOH. Taken together, the deletion of the wildtype allele and duplication of the mutant homologous allele resulted in the elevated MAF of the *GNAQ* mutations.

**Fig. 5:**
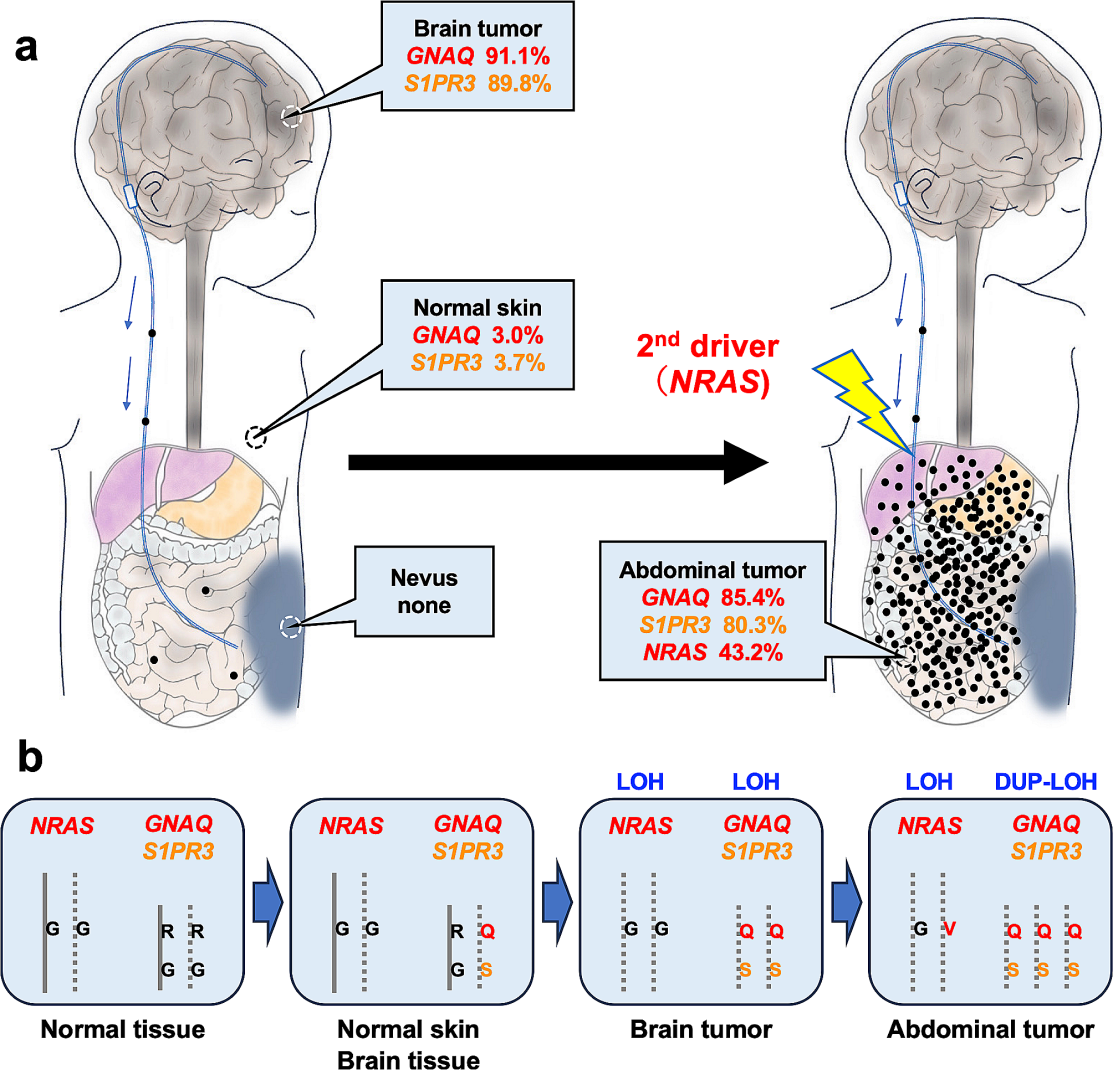
Schematic representation of the distribution of pathogenic driver mutations and clonal lineage in this patient. Distribution and MAF of three pathogenic somatic mutations **(a)**. Additional *NRAS* mutation, playing a role as a second driver, accelerated abdominal tumor growth in this patient. Schematic representation of initiation and progression of tumor in the present case **(b)**. *Abbreviations*: DUP: Duplication; LOH: Loss-of-heterozygosity; MAF: mutant allele frequency

In the present study, we set out to reveal the additional hits required for malignant transformation of leptomeningeal melanomatosis in an NCM patient, by comparing the molecular profile of tissues obtained during surgery and at autopsy, including brain and abdominal tumor, cerebral cortex, and white matter, normal skin, nevus, and kidney (renal medulla). We expected to reveal additional hits in the brain tumor not seen in the nevus. However, somewhat surprisingly, we found that the founding pathogenic mutations were shared in the brain tumor and normal-looking skin obtained at autopsy (Fig. 5a). These mutations were not found in the nevus DNA by WES and ddPCR, but this was thought in part, to be due to low cellularity of the nevus (Fig. 1d and Additional file: Fig. S1a). Also, the nevus tissue was obtained during VP shunt surgery, as opposed to all other samples, which were obtained at autopsy.

Clinically, the progression of leptomeningeal and disseminating lesions was observed. However, MR images and autopsy specimens indicate that the lesions were located almost purely at the surface of the brain and spine. Therefore, the patient was responsive and able to communicate right up to the last minute before her death. Indeed, the autopsy indicated that she did not die of a brain hernia, and the main cause of death was massive ascites restricting the expansion of her lungs. The pathogenic *NRAS* G12V mutation was found only in the abdominal tumor but not the brain tumor. We concluded that additional *NRAS* mutation caused the abdominal tumor to become more aggressive leading to her death. Because the patient presented with severe communicating hydrocephalus leading to a deterioration in the level of consciousness, and considering the patient’s young age, not performing a VP shunt at presentation would not have been an option.

In conclusion, by sampling and molecular analyses of multiple tissues in a NCM patient, we found mutations and CN-LOH responsible for initiation and malignant progression (Fig. 5b). Similar analysis should be done in multiple NCM patients, potentially elucidating the mechanism of malignant progression of leptomeningeal lesions to melanomatosis, hopefully leading to treatment of these patients.

### Electronic supplementary material

Below is the link to the electronic supplementary material.

Supplementary Material 1: Figure S1: Histology of the surgical and autopsy specimens. A small number of melanocytes were scattered in the dermal layer (a). Round to spindle-shaped tumor cells with nuclear atypia filled the subarachnoid space on the brain surface. The proportion of melanin-containing tumor cells was relatively small (b). In immunohistochemistry, the tumor cells were positive for HMB45 (c) and vimentin (d), and negative for S100 (e). MIB-1 labeling index was 10.3% (f). Histology of the brain surface (g) and Sylvian fissure (h) at autopsy. Tumor cells massively infiltrated the brain parenchyma adjacent to the Sylvian fissure via Virchow-Robin space and showed high cellular atypia in the infiltrated area (i). Abbreviations: F: frontal lobe; SF: Sylvian fissure; T: temporal lobe. Scale bars: a-f, i: 50 µm; g: 500µm; h: 200 µm.

Supplementary Material 2: Figure S2: Histology of the abdominal organs. Numerous tumor cells were observed on the serosal surface and infiltrated most of the abdominal organs, including the liver, omentum, diaphragm, and ovaries. Scale bar: 5 mm.

Supplementary Material 3: Figure S3: Allele-specific analysis in non-tumor tissue. Copy number events were not detected in the cerebral cortex, white matter, normal skin, or nevus.

Supplementary Material 4: Table S1: A summary of Primer/Probe mix for ddPCR analysis.

Supplementary Material 5: Table S2: A summary of the number and sites of somatic mutations.

Supplementary Material 6: Table S3: Identification of CNV in the gene bodies of *GNAQ, S1PR3,*  and *NRAS*.  *Abbreviations:*  CNV: copy number variation; LOH: loss-of-heterozygosity

## Data Availability

The datasets acquired during the current study are available from the corresponding author upon reasonable request.

## Abbreviations

CMN: Congenital melanocytic nevi
CN-LOH: Copy-neutral loss-of-heterozygosity
CNS: Central nervous system
CNV: Copy number variation
CSF: Cerebral spinal fluid
ddPCR: droplet digital polymerase chain reaction
HE: Hematoxylin and eosin
MAF: Mutant alleles frequency
NCM: Neurocutaneous melanosis
SNV: Single nucleotide variant
VP: Ventriculo-peritoneal
WES: Whole exome sequencing
