# Supplementary figures and images for: Missense mutation of NRAS is associated with malignant progression in neurocutaneous melanosis
Source: Acta Neuropathol Commun. 2024 Jan 22;12:14. doi: 10.1186/s40478-024-01723-0 (PMC10804483; doi:10.1186/s40478-024-01723-0)

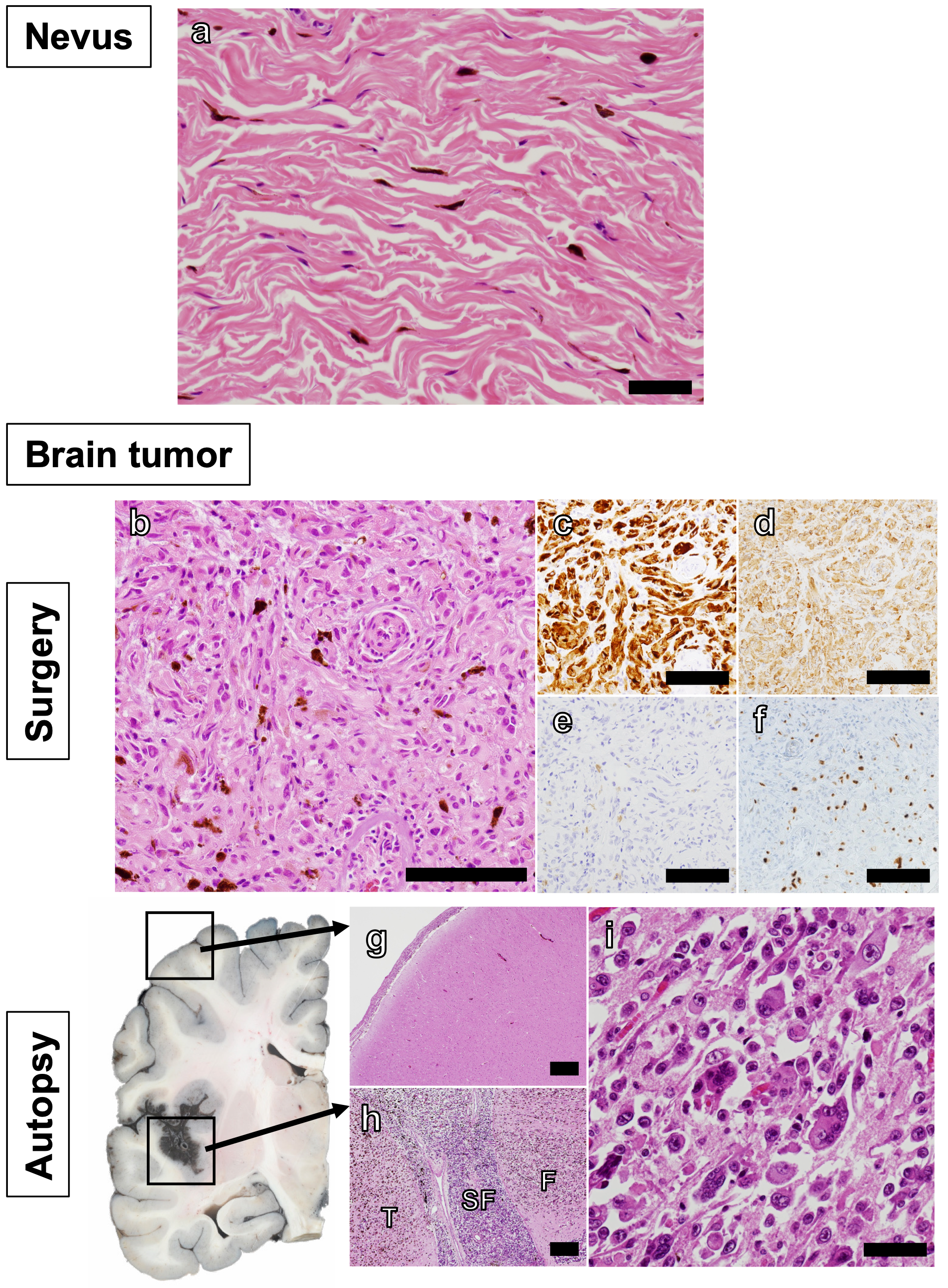

Supplement: Supplementary file 1 — Supplementary Material 1: Figure S1: Histology of the surgical and autopsy specimens. A small number of melanocytes were scattered in the dermal layer (a). Round to spindle-shaped tumor cells with nuclear atypia filled the subarachnoid space on the brain surface. The proportion of melanin-containing tumor cells was relatively small (b). In immunohistochemistry, the tumor cells were positive for HMB45 (c) and vimentin (d), and negative for S100 (e). MIB-1 labeling index was 10.3% (f). Histology of the brain surface (g) and Sylvian fissure (h) at autopsy. Tumor cells massively infiltrated the brain parenchyma adjacent to the Sylvian fissure via Virchow-Robin space and showed high cellular atypia in the infiltrated area (i). Abbreviations: F: frontal lobe; SF: Sylvian fissure; T: temporal lobe. Scale bars: a-f, i: 50 µm; g: 500µm; h: 200 µm. [file 40478_2024_1723_MOESM1_ESM.tiff]

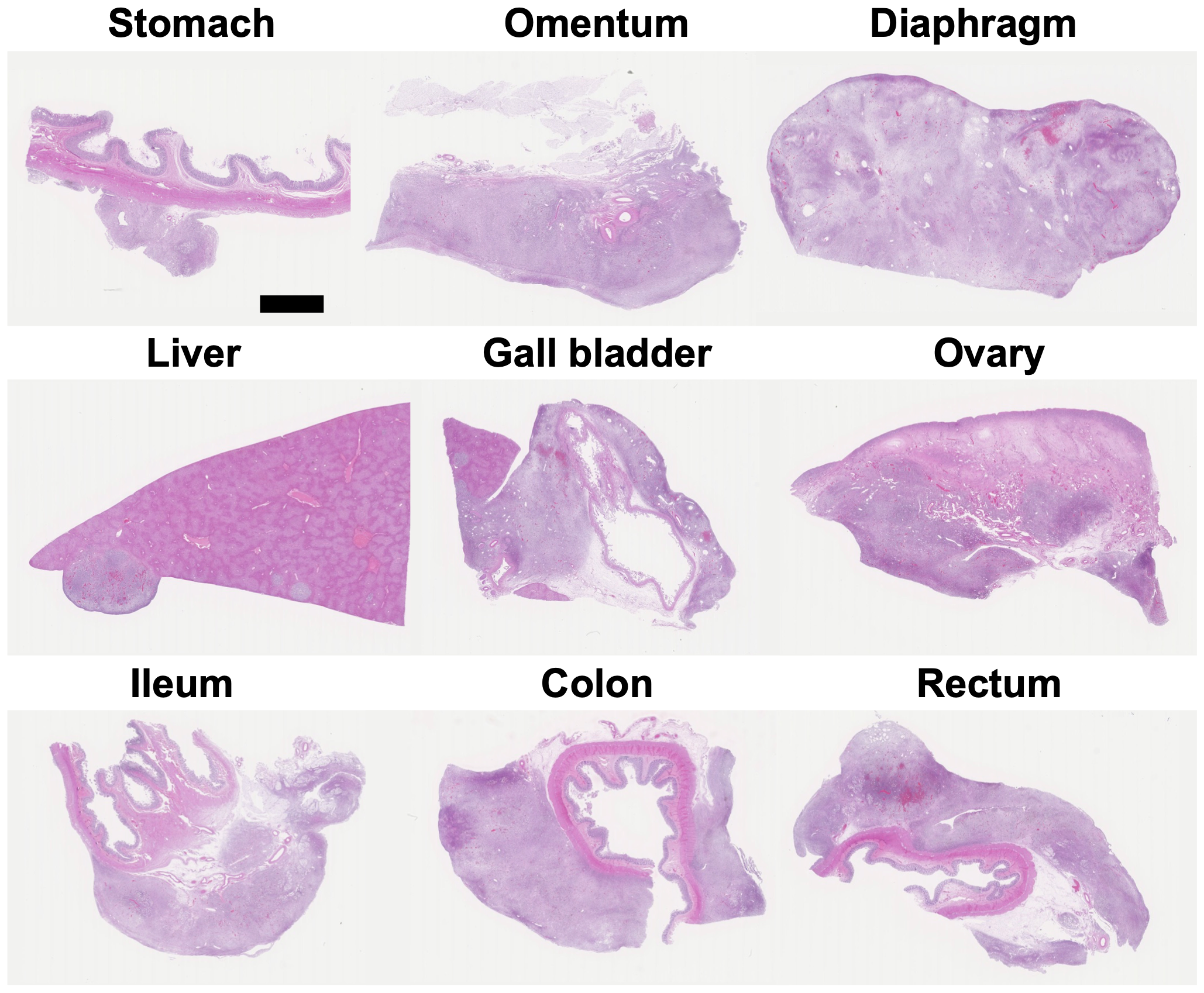

Supplement: Supplementary file 2 — Supplementary Material 2: Figure S2: Histology of the abdominal organs. Numerous tumor cells were observed on the serosal surface and infiltrated most of the abdominal organs, including the liver, omentum, diaphragm, and ovaries. Scale bar: 5 mm. [file 40478_2024_1723_MOESM2_ESM.tiff]

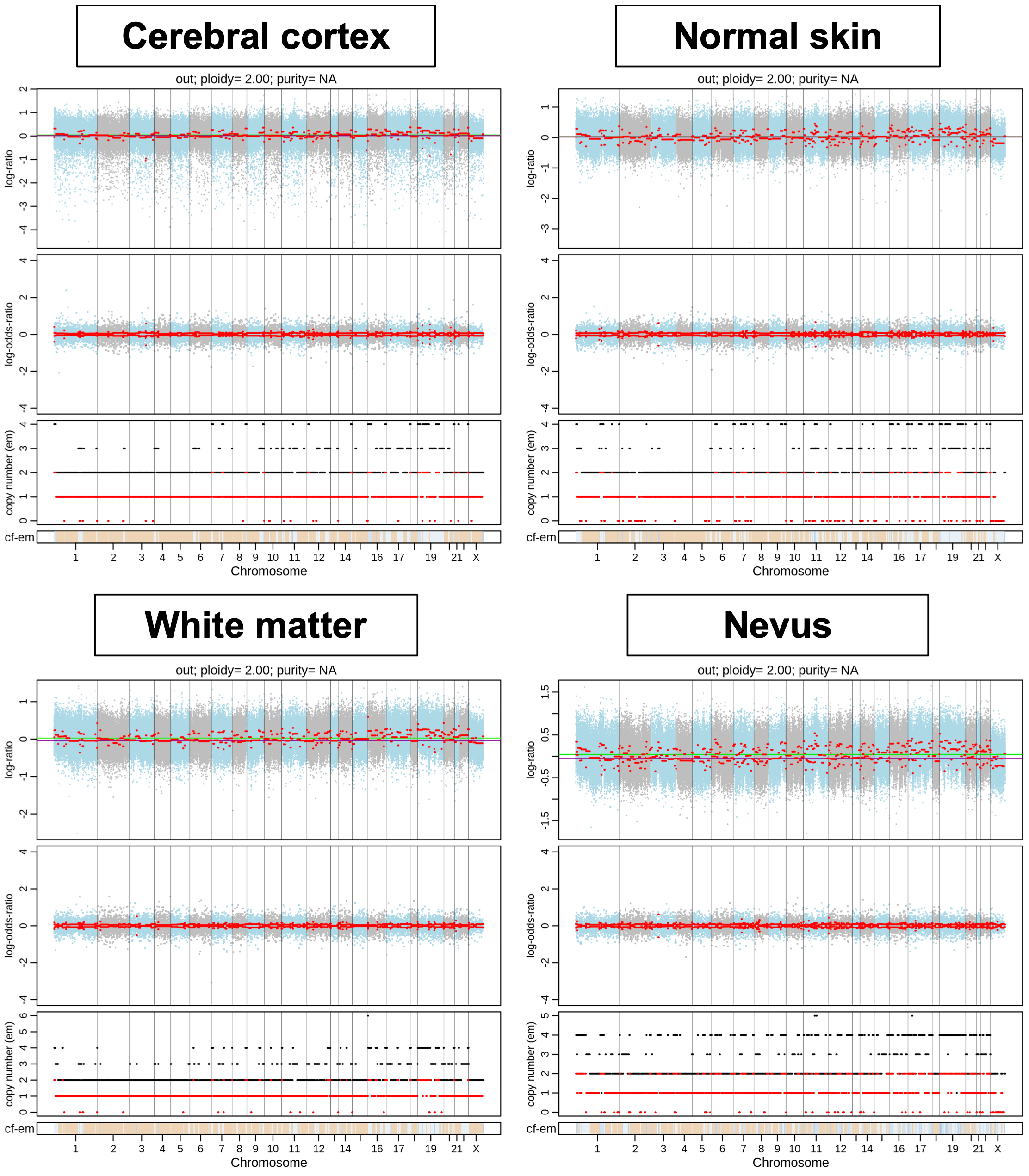

Supplement: Supplementary file 3 — Supplementary Material 3: Figure S3: Allele-specific analysis in non-tumor tissue. Copy number events were not detected in the cerebral cortex, white matter, normal skin, or nevus. [file 40478_2024_1723_MOESM3_ESM.tiff]
